# The Medical Profession and the War

**Published:** 1917-02-10

**Authors:** 


### The Hospital

The Workers' Newspaper of Administrative Medicine and Institutional Life, Administration, National Insurance and Health.

No. 1601, Vol. LXI. SATURDAY, FEBRUARY 10, 1917. PRICE ONE PENNY.

### THE MEDICAL PROFESSION AND THE WAR.

The records of the war, when they come to be written in detail, will include many truths and experiences of interest and importance to members of the medical profession. In the broadest light these experiences have displayed the profession in a relation to the nation and the Army which, prior to the war, had been very imperfectly appreciated. That an army in the field has need of doctors to care for sick and wounded soldiers, just as a civil community requires ready medical assistance to meet the ordinary ills and accidents of life, was manifest to everyone. But beyond this point public opinion hardly advanced. There had, it is true, been a much wider ambition among some of the responsible heads of the Army Medical Service, but such voices hardly reached the public, and even the soldiers were not greatly stirred by them. Events have had a quickening effect in both of these directions. First among these stimulating influences, at least in order of time, has been the enormous demand made on the civil profession to come to the help of the Army. Another factor which may fairly be mentioned is the work so freely undertaken by the voluntary hospitals throughout the length and breadth of the land. The medical and surgical staffs of these institutions have willingly given gratuitous service to help the men wounded and broken in the war, and they have neither asked for official recognition nor expected it. The opportunity has been one for quiet but efficient patriotism, and it has been welcomed in this spirit. Yet in both of the respects here indicated the educational tendency has been a limited one. To the public the work of the doctors, whether on active service in the field or in the hospitals at home, has been concerned with the repair of mischiefs and with the care of individuals. It has thus appeared as the natural and immediate duty of the profession, and though increased in amount and appreciated as of high importance, it has not differed in essence from the workaday function of the practitioner. If the doctors have seemed more than ever necessary, this, it is roughly judged, is because there have been more patients needing their services. The experience has carried its lesson, but the lesson, though set in larger characters, has been similar in nature to that taught by the emergencies of civil life.

The wider educational influence exercised by the medical activities of the war has arisen from a recognition of the capacity of medicine, not merely to care for stricken individuals, but to lessen or prevent the incidence of many of the diseases to which armies in the field are peculiarly liable. That prevention is better than cure is as true for the soldier as it is for the civilian, and the Army chiefs are no doubt fully in sympathy with this humane doctrine. But for Army purposes the doctrine has a more stern application. If only the diseases which have decimated armies in the past can be prevented the number of men kept in the fighting ranks will be correspondingly increased. Given the medical achievement, the increased military efficiency follows as a matter of course. Thus, judged entirely from the soldiers' standpoint, the chief function of the Army doctor, if only it can be realised, is to prevent disease, for by so doing he adds to the numbers of the fighting men. Now it is here that there stands out in peculiarly distinct fashion one aspect of the contribution which medicine has made to the winning of the war. The achievement could not be better summarised than in the words written by Sir Douglas Haig in his last dispatch: "The health of the troops has been most satisfactory, and, . . . there has been an almost complete absence of wastage due to diseases of a preventable nature." No such verdict from a general commanding a vast army in the field has ever before been recorded, and, if rightly regarded, the announcement is more impressive by far than multitudes of figures and statistics and percentages. It records a triumph of a truly sensational character, and its practical meaning in terms of fighting value can hardly be overestimated. Put aside for the moment all the risks and values of those who stand ready to help men stricken on the battlefield; put aside also the hospital services at the base and at home—and there remains what is now announced as a splendid and efficient achievement contributed by medicine to the national cause. That sick and wounded soldiers need efficient doctors is obvious to every eye. But never before has it been written so plainly that the great service which the medical profession can render to the combatant ranks is the prevention of preventable disease.

Necessarily there arises from this result the question—How has this aim been secured? The answer includes two factors. First is the teaching which defined the aim as the one to be attempted, and to no one is the nation more indebted for this teaching than to Sir Alfred Keogh. Not always to attentive listeners, Sir Alfred stuck to his text and expounded his doctrine. He has come back to the Director-Generalship to see adopted in war the ambition which he never failed to urge in days of peace. The second factor in the determination of success has without question been the extension and organisation of scientific medical knowledge. The prevailing character of such extension has been to a larger and larger extent an endeavour to discover the actual causes of various diseases as a necessary first step to their prevention. Laboratory investigations, which to the careless observer seemed concerned rather with high and dry academic doctrine than with the treatment of suffering patients, have come to a full justification, for out of them have developed the methods which have saved our armies from otherwise inevitable perils. A further step to this great issue is the organising and directing work of the Medical Research Committee, which owes so much to the inspiration and efficiency of its secretary, Dr. W. M. Fletcher. The Committee has proved itself a ready and capable instrument for the solution of many of the medical problems which the war has rendered urgent. It has recognised and discovered the experienced worker, and has been able to define the questions at issue, and to place the essential problems in appropriate hands. Work has thus not been left to individual initiative and enthusiasm, but has been ordered and controlled according to a carefully considered scheme. The fruits of the enterprise are valuable beyond words. Great in themselves in relation to the circumstances of the hour, they will stand as an example which will act as an incentive even when men learn war no more.

